# Combating “dread shed”: The impact of overlapping topical and oral minoxidil on temporary hair shedding during oral minoxidil initiation

**DOI:** 10.1016/j.jdin.2024.03.005

**Published:** 2024-03-25

**Authors:** Ambika Nohria, Deesha Desai, Michelle Sikora, Soutrik Mandal, Jerry Shapiro, Kristen Lo Sicco

**Affiliations:** a Ronald O. Perelman Department of Dermatology, New York University Grossman School of Medicine, New York, New York; b University of Pittsburgh School of Medicine, Pittsburgh, Pennsylvania; c New York Medical College, Valhalla, New York

**Keywords:** alopecia, hair loss, oral minoxidil, shedding, side effect, topical minoxidil

## Abstract

**Background:**

Low dose oral minoxidil (LDOM) is a preferred treatment for alopecia due to ease of use and efficacy. While LDOM is typically well tolerated, patients may experience a temporary increase in hair shedding starting treatment, colloquially regarded as “dread shed”. One proposed method to combat this is to overlap therapies by maintaining use of topical minoxidil when initiating LDOM.

**Objective:**

To evaluate the impact of maintaining topical minoxidil when initiating LDOM on “dread shed”.

**Methods:**

We performed a retrospective chart review of patients seen at New York University Langone Health Dermatology from January 1, 2008 to August 1, 2023 prescribed LDOM.

**Results:**

A total of 115 patients met inclusion criteria, of whom 37 maintained use of topical minoxidil when initiating LDOM. Six patients experienced “dread shed” when initiating LDOM, 2 of whom overlapped therapies. We did not find that overlapping therapies had a significant impact on decreasing rates of “dread shed”.

**Limitations:**

Limitations include retrospective design, sample size, and subjective patient-reported assessment of hair shedding.

**Conclusions:**

A total of 5.2% of patients experienced dread shed, which is lower than previously reported in literature. Maintaining topical minoxidil during LDOM initiation does not significantly impact “dread shed”. This remains a significant side effect deserving of further research.

**Capsule Summary**

• This article highlights the side effect of temporary hair shedding after starting low dose oral minoxidil (LDOM). We investigate the impact of overlapping topical minoxidil when starting LDOM.
• Overlapping topical and oral therapy does not significantly impact shedding. Dermatologists may advise patients to discontinue topical therapy when starting LDOM.

## Introduction

Prior to the introduction of topical minoxidil, the treatment options for patients with hair loss remained limited, and primarily focused on cosmetic management. In 1988, the Food and Drug Administration formally approved topical minoxidil for the treatment of androgenetic alopecia (AGA) in males, and later in 1992 for females, marking a dramatic shift in the therapeutic landscape for hair loss disorders.^1^ Now, topical minoxidil is a widely used, efficacious treatment for hair loss that continues to be a cornerstone of medical therapy for many patients. However, to obtain maximum results in both men and women, it must be applied to the scalp twice daily, which can be cumbersome and cosmetically bothersome to many patients.^1^ More recently, low dose oral minoxidil (LDOM) has been used off-label for the treatment of alopecia and is increasingly popular as many patients find it easier to comply with compared to daily application of topical therapy.^2^ Although used at higher doses for its antihypertensive properties, large scale studies have demonstrated LDOM to have a favorable safety profile with low risk of side effects when employed for the treatment of alopecia.^3^ LDOM has demonstrated efficacy as a treatment for hair loss in both men and women. A recent review article reported benefits seen in patients taking doses ranging from 0.5 mg/day to 5 mg/day.^4^ Most frequently, LDOM is employed as treatment for AGA, however, recent research has also demonstrated benefit in patients with other forms of hair loss including telogen effluvium, alopecia areata, and scarring alopecia.^4^

While LDOM is typically well tolerated, 1 notable side effect is a temporary increase in hair shedding after initiation of treatment. This commonly seen paradoxical increase in hair shedding has colloquially become known as “dread shed” and will be referred to as such throughout this article. Dread shed typically begins 2 to 4 weeks after treatment initiation and lasts anywhere from 3 to 6 weeks.^5^^,^^6^ Mechanistically, dread shed is proposed to occur due to a minoxidil induced shortening of the telogen phase of the hair cycle, resulting in the accelerated loss of hairs which would have likely been shed in the coming weeks to months.^7^ This shedding is usually self-limited and the subsequent increase in hair density and diameter typically results in satisfactory results even if patients may face initial losses.^8^^,^^9^ Despite this, rapid hair shedding can be extremely distressing to patients, particularly for those already struggling with hair loss. Research has shown this side effect is unfortunately common and patients may even discontinue treatment because of it.^10^

One proposed method to combat this dread shed is to overlap the use of maintenance topical minoxidil if they present already on this therapy and oral minoxidil when initiating LDOM therapy. The mechanism of topical minoxidil is the same as LDOM and patients may similarly experience a temporary increase in hair shedding when starting topical therapy. However, if patients are already on topical minoxidil, maintaining its use when initiating LDOM may help to combat the occurrence of a second round of shedding when starting oral therapy, as the introduction of minoxidil will not be a new factor in the hair cycle. The interplay between these 2 medication formulations and their role in dread shed remains unclear and warrants further investigation to help guide best clinical practices. In this study, we aimed to evaluate whether continued use of topical minoxidil when starting LDOM impacted the occurrence of a new onset of hair shedding when transitioning to oral therapy.

## Methods

A retrospective analysis was conducted of patients with a diagnosis of alopecia who were prescribed LDOM and seen within the New York University Skin and Cancer Clinic or Faculty Group Practices between January 1st, 2008 and August 28th, 2023. Inclusion criteria included age 18 or older, at least 2 documented office visits, a diagnosis of AGA, and documentation of taking LDOM.

Within our cohort, patients were categorized as those who were using topical minoxidil prior to starting LDOM and were instructed to maintain its use when initiating oral therapy, and those who began LDOM without concurrently already using topical minoxidil. Data obtained included patient demographics, treatment protocols, and patient and physician reported outcomes. Descriptive statistics were used to summarize data. Statistical analysis was conducted using Fisher’s exact test and the statistical significance was set at *P* value <.05. This study was approved by the New York University Institutional Review Board.

## Results

Of 280 charts reviewed, 115 patients met inclusion criteria. Of these, 55% were females with a mean age of 47.25 years. At their initial visit, patients were prescribed 0.625 mg to 2.5 mg LDOM daily, with a median dose of 1.25 mg/day (Table I). Within this cohort, 37 patients (32%) were using topical minoxidil prior to initiating LDOM and were advised to continue topical maintenance therapy when beginning oral therapy. Patients were instructed to maintain topical therapy for an overlap period of at least 1 month when initiating LDOM. The patients that overlapped maintenance topical therapy were 73% females with a mean age of 50.91 years and were prescribed a median daily LDOM dose of 1.25 mg (Table I). Comparatively, 78 patients (68%) were not using topical minoxidil prior to starting oral treatment and thus were not instructed to maintain topical therapy when starting LDOM. These patients were predominantly males (54%) with a mean age of 45.51 years and were prescribed a median daily LDOM dose of 1.25 mg. (Table I). When comparing those that used topical minoxidil to those that did not, there was no significant difference in age or length of time between follow-up visits (*P* = .16, *P* = .75). However, there were significantly more females in the cohort that maintained topical minoxidil (*P* = .01).

**Table I:** Patient demographics

	Overall cohort (*n* = 115)	Topical overlap patients (*n* = 37)	No topical overlap patients (*n* = 78)
Mean age (standard deviation)	47.25 (17.48)	50.91 (19.80)	45.51 (16.12)
Gender	Males, 52 (45%)	Males, 10 (27%)	Males, 42 (54%)
	Females, 63 (55%)	Females, 27 (73%)	Females, 36 (46%)
Median minoxidil dose in mg/day (range)	1.25 (0.625-2.5)	1.25 (0.625-2.5)	1.25 (0.625-2.5)

Within our overall cohort of patients that took LDOM regardless of their use of topical minoxidil, 29.6% endorsed shedding at their initial visit and 19.1% at their first follow-up. There was no statistically significant change in reports of shedding from initial to follow-up visit (*P* = .09). Six patients reported a new onset of shedding after the initial visit that occurred after starting LDOM. These patients were defined as having experienced dread shed with the new introduction of LDOM. The mean length of time between follow-up visits for our overall cohort was 124.9 days (Table II).

**Table II:** Patient reported hair shedding

	Overall cohort (*n* = 115)	Topical overlap patients (*n* = 37)	No topical overlap patients (*n* = 78)
Shedding at initial visit	34 (29.6%)	15 (40.5%)	19 (24.4%)
Shedding at follow-up visit	22 (19.1%)	8 (21.6%)	14 (17.9%)
Change in patients reporting shedding from initial to follow-up visit	12 (10.45%)	7 (18.9%)	5 (6.4%)
New onset shedding after LDOM initiation	6 (5.2%)	2 (5.4%)	4 (5.1%)
Mean length of time between visits (standard deviation)	124.9 days (77.50)	118.5 days (54.03)	127.9 days (86.59)
*P* value for change in shedding	*P* = .091	*P* = .132	*P* = .433

*LDOM*, Low dose oral minoxidil.

Among the 37 patients who overlapped maintenance topical and oral therapy, 40.5% endorsed shedding at their initial visit, and 21.6% endorsed shedding at follow-up. There was no statistically significant change in reports of shedding from initial to follow-up visit (*P* = .13). Within this cohort, 2 patients experienced a new onset of shedding after initiating LDOM, meaning they experienced dread shed. The mean length of time between initial visit and follow-up was 118.5 days (Table II).

Similarly, for the 78 patients who did not overlap topical therapy when starting LDOM, 24.4% endorsed shedding at their initial visit and 17.9% endorsed shedding at follow-up. There was no statistically significant change in reports of shedding from initial to follow-up visit (*P* = .43). Four of these patients experienced dread shed, meaning a new onset of shedding after initiating LDOM. The mean length of time between initial visit and follow-up was 127.9 days (Table II).

We found no statistically significant difference in the change in reported shedding between those who overlapped therapies and those who did not (*P* = .09). Considering dread shed specifically, we found there was no significant association between experiencing dread shed and patients' use of overlapping topical and oral therapy (*P* = .0). Similarly, we found no significant association between rates of dread shed and dose of LDOM prescribed (*P* = .49). Further, there was no significant association between dread shed and gender (*P* = .11) (Table II).

## Discussion

Although minoxidil is considered to be a well-tolerated medication, in both topical and oral formulations, the side effect of dread shed is under-recognized and often inadequately addressed. In our patient cohort, 5.2% of patients reported dread shed, defined as a new onset of shedding after initiating LDOM therapy. Prior literature evaluating tolerability of LDOM have reported rates of increased shedding ranging from 2.4% to 22%.^11^^,^^12^ Considering similar studies on the side effect profile of LDOM report adverse effects such as lightheadedness, edema, and tachycardia occurring at rates less than 2% each, one can appreciate the significance of dread shed as a side effect.

It is well-established that patients who face hair shedding, most commonly as a symptom of AGA or telogen effluvium, suffer from significant psychosocial distress and negative impacts on quality of life.^13^ It is also understood that stress can not only be a result of hair loss but may also be a modifying factor in the progression of alopecia itself.^14^ This creates the potential for a vicious cycle of hair loss that is exacerbated and perpetuated by stress in the context of continued shedding.^14^ Although self-limited increased shedding is not a permanent form of hair loss and most patients will likely regain both the hair loss and more after initiating minoxidil treatment, hair on average grows at a rate of 1 cm per month, meaning that it may take months to years for hair regrowth to reach a cosmetically acceptable level.^15^

Given the scope of this problem, we aimed to evaluate whether there were any associations between maintaining use of topical minoxidil for an overlap period when starting LDOM and levels of hair shedding, in the hopes of developing more management strategies for patients. Our study did not find that maintaining topical minoxidil when initiating LDOM significantly reduced the occurrence of a new onset dread shed when starting oral therapy. This suggests that the continuation of topical minoxidil may not be of significant clinical utility when initiating LDOM. These findings are particularly salient considering prior results demonstrating that the concurrent use of topical minoxidil does not significantly impact hair counts compared to the use of LDOM alone.^16^ We also did not identify any significant changes in reports of dread shed based on LDOM dose, suggesting that higher doses of medication may not result in worsening of this side effect.

The findings of this retrospective chart review have important implications for clinical practice. The daily or twice daily application of topical minoxidil can be laborious and time consuming for patients. The topical solutions or foams can also make hair care difficult for many patients and disrupt their existing hair styling practices. Further, patients may experience side effects including scalp irritation and allergic contact dermatitis.^2^ If the continuation of topical minoxidil when initiating LDOM therapy does not significantly reduce hair shedding, patients may be spared from the aforementioned challenges of topical therapy.

These preliminary results should be interpreted with consideration for the small sample size of patients reporting dread shed, however, our findings suggest that maintaining topical therapy for an overlap period when initiating LDOM does not mitigate the development of dread shed. Further research using larger samples are warranted to more thoroughly evaluate the role of maintenance topical minoxidil when initiating LDOM and to investigate other potential strategies for the management of dread shed.

## Limitations

This study is limited by its retrospective design, limited sample size, and reliance on medical records, which may contain incomplete or missing data. Our study relied on patients' self-reported levels of hair shedding, which is a subjective method of assessment. Additionally, hair shedding is a multifactorial symptom thus it is difficult to definitively conclude the impact of overlapping these medications outside of confounding factors. To limit factors that may contribute to hair shedding including telogen effluvium or ongoing inflammation in the setting of scarring alopecia, we excluded patients with a diagnosis other than AGA. However, additional factors may contribute to shedding which we are unable to control for.

## Conclusion

This study provides valuable insights into the interplay between topical and oral minoxidil. Overlapping maintenance topical minoxidil during oral minoxidil initiation does not appear to significantly impact the occurrence of new onset hair shedding when initiating LDOM. Continuing topical minoxidil when transitioning to LDOM may be of low clinical utility. Dread shed was experienced by only 5.2% of our cohort, which is lower than reported in previous studies. Despite this, dread shed remains a significant side effect which is deserving of further research to better understand and address it. Providers should continue to carefully consider the utility of both topical and oral minoxidil to maximize response to treatment and optimize patient satisfaction.

## Conflicts of interest

Dr Lo Sicco has been an investigator for Regen Lab and is an investigator for Pfizer. Dr Lo Sicco is a consultant for Pfizer and Aquis. Dr Shapiro is an investigator and consultant for Pfizer and is a consultant for Lilly.
